# Variability of ventilation during exercise is greater in adolescents than in adults

**DOI:** 10.14814/phy2.70846

**Published:** 2026-03-27

**Authors:** Ryan Welch, Sarah Casey, John Kolbe, Kevin Ellyett

**Affiliations:** ^1^ The University of Auckland Auckland New Zealand; ^2^ Respiratory Services, Te Whatu Ora Te Toka Tumai Auckland Auckland New Zealand

**Keywords:** adolescents, approximate entropy, exercise, Poincaré, ventilatory control

## Abstract

Adolescence involves rapid physiological changes that may affect ventilatory control. This study used Poincaré and approximate entropy (ApEn) analyses to assess ventilatory variability and randomness during steady‐state exercise in adolescents and adults. Twelve adolescents (10–14 years) and 10 adults (25–35 years) completed 20 min of submaximal cycling at 1.0 W·kg^−1^. The final 15 min were analyzed using Poincaré and ApEn. Continuous variables were expressed as median (interquartile range) and compared by the Mann–Whitney *U*‐test (*α* = 0.05). Adolescents showed greater breathing frequency (BF) standard deviation (SD) 1 4.7 (3.7–5.4) vs. 2.6 (2.5–3.3) 1·min^−1^ (*p* < 0.01), BF SD2 4.9 (4.2–6.8) vs. 3.6 (3.5–4.3) 1 min^−1^ (*p* = <0.05) and minute ventilation (V̇_E_) relative to body weight SD1 0.02 (0.02–0.03) vs. 0.01 (0.01–0.02) L·min^−1^·kg (*p* = <0.01). ApEn was also higher in adolescents for V̇_E_ 1.23 (1.20–1.32) vs. 1.01 (0.93–1.25) (*p* = <0.05), BF 1.26 (1.24–1.33) vs. 1.17 (1.01–1.18) (*p* < 0.01) and tidal volume 1.33 (1.26–1.37) vs. 1.23 (1.14–1.27) (*p* < 0.05). This study suggests that adolescents showed greater ventilatory variability and randomness compared to adults, likely reflecting developmental plasticity and immature ventilatory control.

## 1 INTRODUCTION

The inputs controlling ventilation during exercise arise from peripheral and central chemoreceptors, chest wall and pulmonary receptors, mechanoreceptors, vagal afferents, and respiratory and non‐respiratory central mechanisms. The control of exercise ventilation, which comprises feedback and feedforward mechanisms, is challenged during adolescence by physiological changes (respiratory and neurologic) and other factors such as hormonal status and changes to body habitus. Neural plasticity may be necessary to facilitate the maturation of the exercise ventilatory control during adolescence, a period characterized by rapid physiological change and growth (Mitchell & Babb, 2006). While there have been suggestions that healthy, active children and adolescents have variable breathing patterns during exercise (Nourry et al., 2005), breathing patterns during exercise in this population remain understudied and poorly understood.

In the analysis of ventilation, there is increasing awareness that nonlinear analysis techniques such as Poincaré and approximate entropy (ApEn) analysis may complement the information obtained from linear methods, potentially furthering our understanding of breathing patterns during exercise in both health and pathology. Poincaré analysis is a geometric and non‐linear technique used to quantify the short‐ and long‐term variability of time series data (Hsu et al., 2012; Satti et al., 2019). Poincaré analysis has shown increased ventilatory variability in various clinical populations, including greater breathing frequency (BF) variability in heart transplant candidates with exercise oscillatory ventilation (Welch et al., 2021), higher variability in heart failure (HF) and COPD plus HF compared to COPD alone (Fernandes et al., 2022), and elevated minute ventilation (V̇_E_) variability during exercise in COPD patients breathing heliox versus room air (Gomes et al., 2023). ApEn is a measure used to quantify the degree of randomness in a data series (Pincus, 1991). ApEn has been used to characterize the degree of randomness of ventilation in health (Pincus, 1991), asthma (Veiga et al., 1985), subjects with hyperventilation and panic disorder (Caldirola et al., 2004; Yeragani et al., 2002), breathing pattern disorder (Bansal et al., 2018) and dysfunctional breathing post COVID‐19 infection (Samaranayake et al., 2023).

Some studies have applied non‐linear analysis techniques to an entire cardiopulmonary exercise test (CPET); however, the influence of variable exercise test durations between subjects is a significant limitation (Bansal et al., 2018; Samaranayake et al., 2023). Furthermore, the constantly changing workload profile of a CPET alters breathing patterns throughout, providing small samples of stable data for analysis. Prolonged, submaximal steady‐state exercise addresses these limitations by providing a stable workload profile and sufficient sample to apply Poincaré and ApEn analysis.

The objective of this study was to use Poincaré and ApEn analysis to quantify and compare the variability and randomness of ventilation during submaximal steady‐state exercise between healthy physically active adolescent and young adult subjects. It was hypothesized that Poincaré and ApEn analysis variables are higher in the adolescent group compared to the adult group.

## 2 METHODS

### 2.1 Ethics

This study was conducted in accordance with the principles outlined in the World Medical Association's Declaration of Helsinki and adhered to all relevant ethical guidelines for research involving human participants. Institutional approval from the Auckland District Health Board research review committee (A + 8144) and ethics approval from the Health and Disability Ethics Committees (18/NTB/94) were obtained. Subjects aged ≥16 years provided written informed consent and subjects aged <16 years provided assent and their parent/caregiver provided written informed consent. Informed consent for publication of individual's data was obtained, and all identifying information was removed.

### 2.2 Subjects

Two groups were recruited: the adolescent group (target *n* = 10, 5 M and 5 F) aged 10–14 years and the adult group (target *n* = 10, 5 M and 5 F) aged 25–35 years. Participants in this study were healthy, physically active, had a body mass index (BMI) below 30 kg m^2^, were not on inhaled therapy, and were non‐smokers and non‐e‐cigarette users. Medical histories were reviewed through interviews, and individuals were excluded if they had any diagnosed respiratory, metabolic, or cardiovascular conditions, or musculoskeletal conditions that might impair their ability to undertake exercise. The justification for the age range of the adult group was to ensure that lung volume growth had peaked, and respiratory mechanics were therefore relatively stable.

### 2.3 Protocol

Subjects completed a single study visit consisting of spirometry followed by a submaximal steady‐state exercise test.

### 2.4 Spirometry

Spirometry was performed in the seated position using a screen pneumotach (Vyaire Medical) via a mouthpiece (SureGard filter, Bird Healthcare) while wearing a nose clip (Grip Nose Clips, Bird Healthcare). Spirometry was performed to the American Thoracic Society/European Respiratory Society standards, and *z*‐scores were determined by the global lung initiative reference equations (Graham et al., 2019; Quanjer et al., 2012).

### 2.5 Steady‐state exercise test

The steady‐state exercise test was performed on a cycle ergometer (Ergoline Via Sprint 150p). Unedited breath‐by‐breath data were sampled by a metabolic cart (Vyntus CPX, Jeager, SentrySuite software, CareFusion) via a facemask (Hans Rudolph). It was emphasized that the subject did not talk during the exercise test and continued cycling until they were told to stop. The researcher did not engage with the subject during the test, nor was verbal or non‐verbal encouragement given. Three minutes of baseline data were collected while the subject was seated on the cycle ergometer with the monitoring equipment on. This was followed by 20 min of steady‐state cycling maintaining a cadence of 60 rpm at a workload of 1.0 Watt per kilogram of body weight. The workload was selected to elicit a target heart rate of 60% age predicted maximum. The rationale for this workload was that it would be sufficient to remove the conscious control of breathing without exceeding the ventilatory threshold. The initial 5‐min of exercise was not analyzed, and this allowed the subject to reach steady‐state exercise. In healthy subjects during moderate intensity exercise, V̇_O2_ kinetics, ventilation, cardiac output, and heart rate have been shown to reach steady‐state within 3‐min of exercise onset (Barstow, 1994; Fletcher et al., 2001). The final 15‐min of exercise data were analyzed (as explained below); this allowed an 8‐min familiarization period (3‐min baseline and 5‐min to reach steady‐state) for the subject to become accustomed to the face mask and cycle ergometer prior to the analysis window.

### 2.6 Non‐linear analysis of breathing patterns

Breath‐by‐breath BF, V_T_ and V̇_E_ data were exported from the CPET and used for Poincaré analysis. BF has a mass exponent of −0.25 and is less affected by morphology and body mass as opposed to V_T_ and V̇_E_ which have mass exponents of 1.0 and 0.81, respectively (Dejours, 1975). As such, V_T_ was normalized to FVC (V_T_/FVC), and V̇_E_ was normalized to body weight (V̇_E_/kg) to allow between subject comparisons. Poincaré plots were created by plotting the value of the respective respiratory parameter of the current breath (*n*) against the value of the immediately following breath (*n* + 1). The line of identity was drawn and the standard deviation 1 (SD^1^) and standard deviation 2 (SD^2^) were calculated as described by other authors, see below (Bien et al., 2004; Brennan et al., 2001; Soni & Muniyandi, 2019):

$$SD^{1}=\frac{1}{2}\;\mathrm{SDS}D^{2}$$

$$SD^{2}=2\mathrm{SDB}B^{2}-\frac{1}{2}\mathrm{SDS}D^{2}$$

where SD is the standard deviation and BB is the time‐course of the breath‐to‐breath measure. $SD^{1}$ is defined as the dispersion of data points perpendicular to the line of identity through the centroid of the plot and is a descriptor of breath‐to‐breath or short‐term variability. SD^2^ describes the dispersion of points along the line of identity and reflects the long‐term variability of the signal.

ApEn was used to determine the randomness of V̇_E_, BF, and V_T_ using methods described previously (Pincus, 1991). The *m*‐value (length of sequences to be compared) was set at 2 and the *r‐*value (tolerance threshold for accepting similar patterns between two segments) was set at 0.2. When data contain repetitive and predictable patterns the ApEn value is low (tends towards zero) and when data are more random or unpredictable the ApEn value is higher. ApEn was calculated as per the equations below:

$$C_{\mathrm{im}}\;\left(r\right)=\frac{n_{\mathrm{im}}\left(r\right)}{N-m+1}$$

$$\text{ApEn}\left(S_{N},m,r\right)=\text{In}\frac{C_{m}\left(r\right)}{C_{m}+1\left(r\right)}$$

where *r*: similarity criterion; *n*_im_(*r*): number of patterns that are similar with *r*; *m*: pattern length; *S*_*N*_: sequence of *N* measurements; *C*_*m*_(*r*): mean of all *C*_im_(*r*) values.

### 2.7 Statistics

Continuous data was presented as median and (interquartile range). The Mann–Whitney *U*‐test compared continuous data between the groups. Alpha was set at 0.05.

## 3 RESULTS

The adolescent group consisted of 12 subjects (5 males and 7 females) with a median age of 14 (13–14) years and median BMI of 19.8 (17.1–21.7) kg·m^2^. Ten subjects (5 males and 5 females), median age 32 (28–33) years with a median BMI of 22.9 (22.0–24.1) kg·m^2^ formed the adult group. Spirometry results were within the normal range for all subjects (Table 1). There were no between‐group differences in FEV_1_ and FVC z‐scores (Table 1), but the FEV_1_/FVC *z*‐score was higher (*p* = <0.01) for the adolescent group 0.18 (−0.32–0.57) compared to the adult group −0.70 (−1.03 – −0.39).

**TABLE 1:** Spirometry and steady‐state exercise data.

	Adolescent group (*n* = 12)	Adult group (*n* = 10)
Spirometry		
FEV_1_ (L)	2.93 (2.50–3.28)	3.43 (3.25–4.85)*
FEV_1_ *z*‐score	0.07 (−0.49–0.33)	−0.29 (−0.30 to −0.16)
FVC (L)	3.19 (2.70–3.68)	4.33 (4.10–6.16)*
FVC *z*‐score	−0.03 (−0.84–0.34)	0.33 (−0.25–0.49)
FEV_1_/FVC (%)	90 (86–92)	79 (78–81)*
FEV_1_/FVC *z*‐score	0.18 (−0.32–0.57)	−0.70 (−1.03 to −0.39)*
Steady‐state exercise		
V̇_O2_ (mL⸱min^−1^⸱kg^−1^)	19.0 (17.8–20.2)	18.0 (17.8–18.3)
V̇_E_/kg (L⸱min^−1^⸱kg^−1^)	0.55 (0.51–0.59)	0.51 (0.44–0.53)
HR (bpm)	128 (107–134)	123 (108–135)
HR (% max predicted)	62 (52–65)	63 (57–72)
V_T_ (L)	0.86 (0.77–1.06)	1.67 (1.41–1.87) *
V_T_/FVC (%)	28 (25–29)	35 (32–39) *
BF (1⸱min^−1^)	31 (28–34)	22 (18–25) *
V̇_E_/VCO_2_	26 (25–27)	28 (26–30)
V̇_E_/VO_2_	24 (23–25)	26 (25–26)
PetCO_2_ (kPa)	5.04 (4.91–5.16)	4.88 (4.65–5.41)
PetO_2_ (kPa)	14.29 (14.05–14.41)	14.33 (14.18–14.67)

*Note*: Data presented as median (interquartile range).

Abbreviations: BF, breathing frequency; FEV_1_, forced expired volume in the first second; FVC, forced vital capacity; HR, heart rate; PetCO_2_, end tidal partial pressure for carbon dioxide; PetO_2_, end tidal partial pressure for oxygen; V̇_E_/kg, minute ventilation relative to body weight; V̇_E_/VCO_2_, ventilatory equivalent for carbon dioxide; V̇_E_/VO_2_, ventilatory equivalent for oxygen; V̇_O2_, oxygen uptake; V_T_, tidal volume.

* *p* < 0.05.

The groups were well matched for exercise intensity and there were no between group differences for percent of maximum predicted heart rate (HR), or V̇_O2_ and V̇_E_ relative to body weight (Table 1). Compared to the adult group, on average the adolescent group had a smaller V_T_/FVC 28 (25–29) vs. 35 (32–39) % (*p* = <0.01) and higher BF 31 (28–34) vs. 22 (18–25) 1 min^−1^ (*p* = <0.01). There were no differences in ventilatory equivalents or end tidal partial pressures between the adolescent and adult groups (Table 1).

**FIGURE 1:**
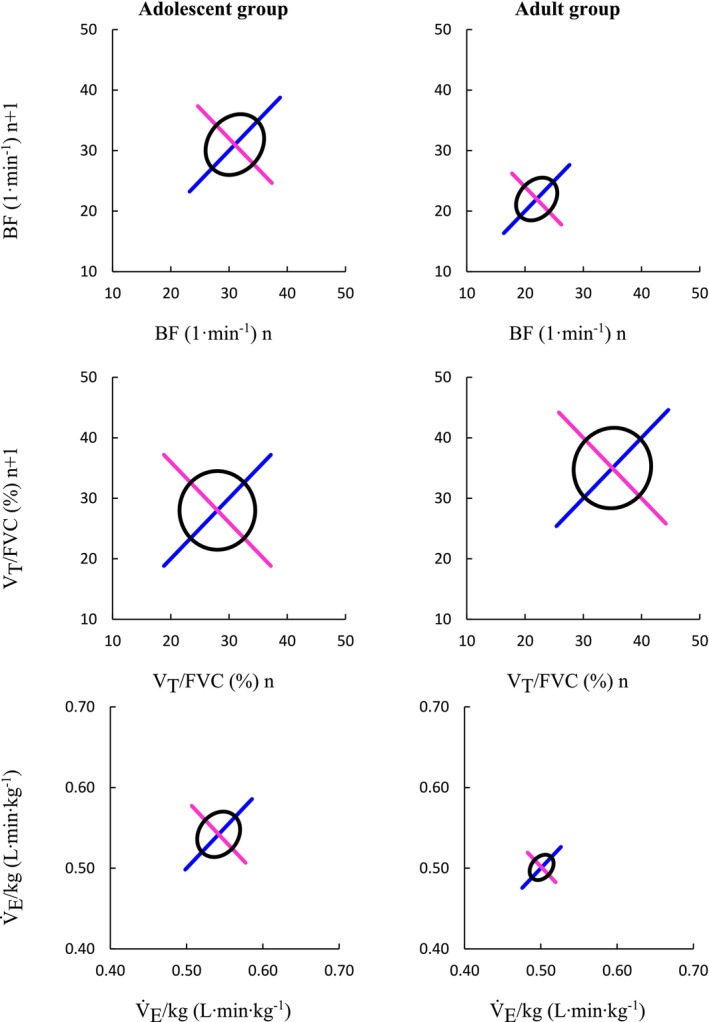
Group average breathing frequency, and relative V_T_ and V̇_E_ Poincaré plots for the adolescent and adult subjects. The black ellipse is created using SD1 (magenta) and SD2 (blue) values. The magenta SD1 line is 2*SD1 and the blue SD2 line is 2*SD2. BF, breathing frequency; V̇_E_/kg, minute ventilation relative to body weight; V_T_/FVC, tidal volume relative to forced vital capacity.

Compared to the adult group, the adolescent group had significantly higher BF SD1 4.7 (3.7–5.4) vs. 2.6 (2.5–3.3) 1 min^−1^ (*p* < 0.01), BF SD2 4.9 (4.2–6.8) vs. 3.6 (3.5–4.3) 1 min^−1^ (*p* = <0.05), and V̇_E_/kg SD1 0.02 (0.02–0.03) vs. 0.01 (0.01–0.02) L·min^−1^·kg (*p* = <0.01) (Figure 1). There were no differences between the adolescent and adult groups for V_T_/FVC SD1 6.5 (4.7–7.9) vs. 6.5 (5.0–9.1) % (*p* = 0.59), V_T_/FVC SD2 6.5 (4.6–9.2) vs. 6.8 (5.7–8.4) % (*p* = 0.70), or V̇_E_/kg SD2 0.03 (0.02–0.04) vs. 0.02 (0.01–0.02) L·min^−1^·kg (*p* = 0.06) (Figure 1).

**FIGURE 2:**
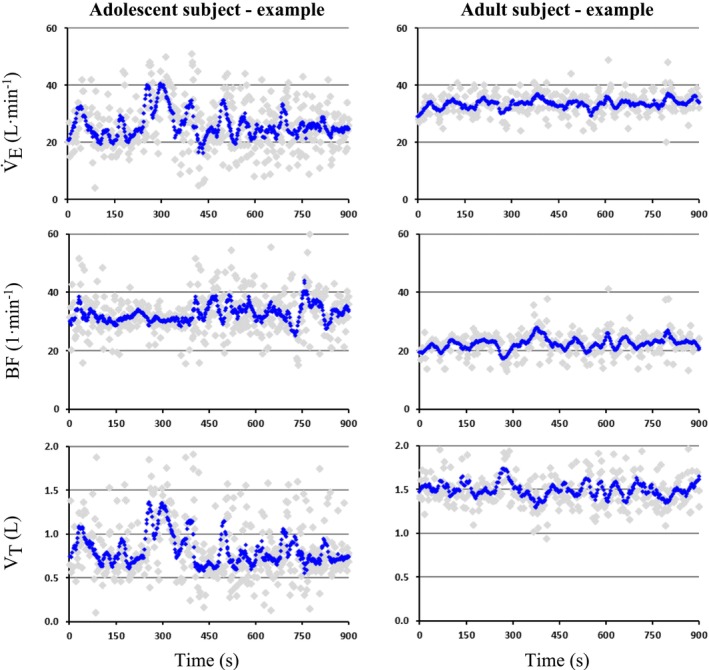
Examples of the ventilatory response during steady‐state exercise. The figures provide an example of the ventilatory response of individual subjects from the adolescent group (left column) and adult group (right column). The top row plots V̇_E_, the middle row is BF, and the bottom row shows V_T_. The light gray points are the breath‐by‐breath data, and the blue points are 8‐breath moving average data. Informed consent for publication of the participant's data was obtained, and all identifying information has been removed. BF, breathing frequency; V̇_E_, minute ventilation; V_T_, tidal volume.

ApEn was significantly higher for the adolescent group compared to the adult group for V̇_E_ 1.23 (1.20–1.32) vs. 1.01 (0.93–1.25) (*p* = <0.05), BF 1.26 (1.24–1.33) vs. 1.17 (1.01–1.18) (*p* < 0.01) and V_T_ 1.33 (1.26–1.37) vs. 1.23 (1.14–1.27) (*p* < 0.05). Figure 2 provides an example of the ventilatory response of the individual from the adolescent group and adult group with the median ApEn V̇_E_ value.

## 4 DISCUSSION

In keeping with our hypothesis, Poincaré and ApEn analysis suggested that adolescent subjects had greater variability and randomness of ventilation during steady‐state exercise compared to adults. We propose that these age‐related differences in variability and randomness of breathing patterns observed in the adolescent subjects reflect the maturing of ventilatory control during adolescence, a period of relatively rapid changes to respiratory system characteristics and the neurological control of ventilation.

### 4.1 Stable vs. unstable ventilatory control system

The greater variability and randomness of ventilation observed in the adolescent subjects should not be viewed as abnormal. In both groups, the ventilatory response during steady‐state exercise may be regarded as representing a “stable” respiratory control system as the average V̇_E_ was relatively constant throughout exercise (Khoo, 2000). However, the significantly greater breath‐to‐breath variability in ventilatory parameters in the adolescent group compared to the adult group likely represents a “less stable” respiratory control system (Khoo, 2000).

Using a simplified model of the chemoreflex control of ventilation, PaCO_2_ is regulated by a negative‐feedback loop, where fluctuations in PaCO_2_ stimulate chemoreflexes to produce ventilatory adjustments which act to restore PaCO_2_ to equilibrium levels. Due to circulatory and processing delays, this response is not instantaneous, leading to an overcorrection which acts to perturb the system in the opposite direction resulting in oscillations in ventilation (Khoo, 2000). The period and amplitude of normally occurring oscillations in ventilation are determined by the plant gain and controller gain of the ventilatory control system. In this context, the plant gain represents the amplification with which ventilatory changes are translated into changes in PaCO_2_, and the controlled gain is the strength of the chemoreflex response. The loop gain (sum of plant gain and controller gain) determines the stability of the system, where a higher loop gain reflects a less stable system. In the context of a negative feedback control of exercise ventilation, a less stable control system will produce a more variable ventilatory response (Khoo, 2000). The respiratory control system does not operate in isolation, and ventilatory variability can also be influenced by other interacting organ systems or from inputs from the cerebral cortex, which allows behavioral and volitional influence on ventilation (Khoo, 2000). A degree of ventilatory variability is normal during exercise. However, the findings of this study suggest that ventilatory variability is influenced by age.

### 4.2 Potential mechanisms for greater variability and randomness of ventilation in adolescents

This observational study cannot identify the mechanisms responsible for the greater variability and randomness of ventilation observed in the adolescent group, but we speculate that it reflects a learning process during adolescence where subjects are exploring different ventilatory solutions to achieve the most mechanically efficient alveolar ventilation. Adolescence is characterized by a period of rapid physical and neurological development, and hormonal, psychosocial, and systemic changes, all of which may affect respiratory system characteristics and the ventilatory control systems during exercise. We propose that during adolescence, a maturation and plasticity of ventilatory control occurs as optimal solutions to ventilatory demands are explored, which manifests as variable and random breathing patterns during exercise. Although hormonal, psychosocial, and systemic changes are acknowledged, the following discussion will focus on respiratory and central nervous system changes during adolescence that may influence these findings.

### 4.3 Changes to respiratory system characteristics

From a respiratory mechanical perspective, adolescence is associated with greater trunk versus limb growth, leading to increased lung volume and airway caliber (Becklake & Kauffmann, 1999; Quanjer et al., 2012; Rosenthal & Bush, 2002). The changes in lung volume and airway caliber do not necessarily occur in unison during adolescence. This is termed dysanaptic growth and is reflected by the deviation in FEV_1_/FVC ratio observed in the GLI spirometry reference data (Quanjer et al., 2010, 2012). Pre‐puberty lung volumes are similar between boys and girls, but post‐puberty males have larger lung volumes than females due to greater width and height of the thorax (DeGroodt et al., 1988). Peak velocity for expired flow rates occurs ~12–14 months after peak velocity for height and coincides with increases in respiratory muscle power and muscle hypertrophy (Hibbert et al., 1995; Merkus et al., 1993). The diaphragm increases in both length and width, resulting in increased forced development and altered function. The net result is a period during adolescence characterized by staggered changes in thorax dimensions, lung volume, airway caliber, and respiratory muscle strength. Considering the control of exercise ventilation relies on feedforward and feedback mechanisms, these mechanical and morphologic changes in respiratory system properties are likely to present an ongoing challenge to the control of ventilation during exercise in adolescents.

### 4.4 Central neural changes affecting the control of exercise ventilation

Occurring concurrently with the physiological changes of the respiratory system, the neurological control systems regulating the ventilatory response during exercise are subject to change and development during adolescence as individuals transition from childhood to adulthood. During childhood and adolescence, the brain is a highly plastic organ and undergoes significant physiological and organizational changes (Mitchell & Babb, 2006). There is a gradual increase in brain volume and simultaneously a pruning of excess neural connections to form more efficient pathways, increased myelination and alterations in the vasculature leading to the development of cerebral blood flow (CBF) (Leung et al., 2016). Modulation and plasticity are important during the adolescent period to adjust and modify the exercise ventilatory response as conditions change during the transition from childhood to adulthood (Mitchell & Babb, 2006).

The CBF responses to changes in PaCO_2_ represent a vital homeostatic function that regulates brain pH levels and influences respiratory drive via central chemoreceptors (Ogoh, 2019; Tarumi & Zhang, 2018). The response of CBF to changes in PaCO_2_ is defined as cerebrovascular reactivity to CO_2_ (CVR) and reflects the vasculature's ability to accommodate changes in blood flow demand in the brain (Ogoh, 2019). Recent studies have shown CVR and CBF are age‐dependent and undergo considerable development between childhood and adulthood (Leung et al., 2016; Talbot et al., 2023; Tallon et al., 2020; Weston et al., 2021).

A cross‐sectional study by Leung et al. (2016) reported the development of CVR in a group of healthy subjects (*n* = 34) aged 9–30 years (Leung et al., 2016). The authors showed that CVR gradually increased with age during childhood and peaked at 14.7 years, after which there was a negative correlation between CVR and age (Leung et al., 2016). Children have higher levels of resting cerebral perfusion which may limit their capacity for further vasodilation, that is, lower cerebrovascular reserve and CVR (Leung et al., 2016). The blunted CVR of children compared to adults may also relate to the elevated ventilatory response to hypercapnia that has been reported in children (Brischetto et al., 1984). In keeping with these results, Tallon et al. (2020) found that the middle cerebral artery mean velocity (MCAv) time constant was faster in adults compared to children in response to hypercapnia which suggests an attenuated CVR response in children (Tallon et al., 2020). During exercise, the acute change in MCAv is attenuated in children when compared to adolescents and adults (Weston et al., 2021). The adolescent group recruited for the current study represent a cohort transitioning from childhood to adulthood, a period characterized by changes in CBF, CVR and sensitivity to CO_2_ (Leung et al., 2016; Talbot et al., 2023; Tallon et al., 2020; Weston et al., 2021). These changes may influence the ability of central chemoreceptors to rapidly respond to changes in PaCO_2_ which may manifest as the variable and random breathing patterns observed in the adolescent subjects.

Age related changes in the ventilatory response to CO_2_ have been described by previous studies (Brischetto et al., 1984; Cooper et al., 1987; Nagano et al., 1998; Springer et al., 1989). Compared to older adults, in normal children during exercise ventilation is greater in response to hypercapnia (Brischetto et al., 1984). Studies suggest that during exercise PaCO_2_ increases and V̇_E_/VCO_2_ slope decreases with age (Cooper et al., 1987; Giardini et al., 2011; Nagano et al., 1998; Weston et al., 2021). Collectively, these studies suggest age‐related changes in exercise ventilatory control characterized by a lower PetCO_2_ set point in children compared to adolescents and adults. The results of this study showed that on average there were no differences in V̇_E_/kg^−1^, ventilatory equivalents or end tidal partial pressures between adolescent and adult subjects during steady‐state exercise. It may be inferred that the adolescent subjects had a similar PaCO_2_ set point and ventilatory efficiency (i.e., same ventilatory equivalents for CO_2_ and O_2_) as the adults during exercise. While the relative ventilatory response (V̇_E_/kg^−1^) was the same between the adolescent and adult groups, the breathing strategy of the adolescent group was different as reflected by higher BF, lower V_T_/FVC and greater variability and randomness of ventilatory response. We suggest that these findings demonstrate the maturation of ventilatory control in the transition from childhood to adulthood.

### 4.5 Normal ventilatory response vs. dysfunctional breathing

The more variable and random breathing patterns observed in the adolescent group may be mistaken for dysfunctional breathing. According to a European Respiratory Society review “dysfunctional breathing is a term describing a group of breathing disorders in patients where chronic changes in breathing pattern result in dyspnoea and often non‐respiratory symptoms in the absence of, or in excess of, organic respiratory disease” (Boulding et al., 2016). While there is no consensus on the definition or gold standard diagnostic criteria of dysfunctional breathing, it is essentially a blanket term for a cluster of breathing patterns that are abnormal, inefficient and result in dyspnoea. To diagnose abnormality (i.e., dysfunctional breathing) there needs to be a clear understanding of normality. Historically, most exercise physiology studies were performed using young adult male subjects, therefore our understanding of the normal ventilatory response in adolescents is rudimental at best (Sheel et al., 2004). It is our contention that the greater variability and randomness of ventilation during exercise in the adolescent subjects does not reflect dysfunctional breathing but rather the normal maturating of ventilatory control. While this is the first study to objectively describe the ventilatory response during exercise in adolescent subjects using non‐linear techniques, unstable/random breathing patterns in healthy children have previously been mentioned in the literature (Nourry et al., 2005).

The ApEn values for the adolescent and adult subjects in this study are higher than previously reported for healthy subjects and are similar to those reported in subjects with dysfunctional breathing (Bansal et al., 2018; Samaranayake et al., 2023). Bansal et al. (2018) reported that ApEn >0.88 for V̇_E_ had high sensitivity (70%) and specificity (87%) for dysfunctional breathing (Bansal et al., 2018). In our cohort, ApEn for V̇_E_ was 1.23 (IQR: 1.20–1.32) in the adolescent group and 1.01 (IQR: 0.93–1.25) in the adult group, which would argue for the presence of dysfunctional breathing according to the Bansal et al. (2018) criteria. However, methodological differences between Bansal et al. (2018) and the current study need to be considered. Compared to our subjects, the Bansal et al. (2018) cohort were older (age ~50 years). Aging is associated with increased airflow obstruction, smaller absolute lung volumes and reduced expired flows which theoretically reduces breathing pattern variability and would result in lower ApEn values. Bansal et al. (2018) applied ApEn analysis to an entire incremental CPET whereas this study used prolonged submaximal steady‐state exercise. For the dysfunctional breathing pattern group in the Bansal et al. (2018) study, the CPET duration was approximately a third lower compared to the controls (Bansal et al., 2018). The influence of variable CPET duration is an important factor as ventilatory variability is theoretically lower between ventilatory threshold and peak exercise compared to pre‐ventilatory threshold. The current study shows that age and exercise intensity are important factors when using non‐linear techniques in the analysis of the exercise ventilatory response.

### 4.6 Strengths, limitations, and future direction

The strength of this study was that prolonged submaximal steady‐state exercise at a standardized workload was used, which removes the influence of changing workload profile on breathing patterns and provides a larger sample of data for non‐linear analysis techniques.

There are several limitations of this study that should be acknowledged. A familiarization session was not conducted for this study. Conscious control of the exercise ventilatory response may have influenced these findings, particularly for the adolescent group who may have found it novel to exercise in a respiratory physiology laboratory whilst wearing a face mask. The influence of the conscious control of exercise ventilatory response was likely reduced by the familiarization period (3‐min baseline and 5‐min to reach steady‐state) with the final 15‐min of submaximal steady‐state exercise used as the analysis window. Furthermore, the workload of 1 W kg^−1^ was specifically selected to elicit a target HR of ~60% heart rate reserve and we deemed it sufficient to reduce the conscious control of exercise hyperpnoea while remaining below ventilatory threshold.

While consistent with many exercise physiology studies, the sample size was low and these findings should be confirmed by a larger study. Consequently, sex‐differences could not be assessed. There are sex‐based differences in the development of the respiratory system during adolescence which may result in greater variability and randomness of breathing patterns in males compared to females. Larger samples should be collected to determine if sex‐based differences in exercise breathing pattern variability and randomness occur during the adolescent period.

Non‐linear analysis techniques require relatively large samples of data. Early recommendations were that 1000 data points are required for ApEn analysis (Pincus, 1991) but more recently it has been shown that for biological time series data smaller samples (200 data points) may be appropriate (Yentes et al., 2013). A pragmatic decision of 15‐min of exercise duration was made based on the likely duration of exercise that could be maintained by the adolescent subjects, and this duration was considered sufficient to apply ApEn and Poincaré analysis.

Future studies should recruit younger children (6–10 years) and older adults (>50 years) and apply non‐linear analysis techniques to assess the steady‐state exercise breathing patterns across the lifespan. Finally, future studies should investigate the mechanisms of the greater variability and randomness of ventilation during exercise in adolescent subjects.

## 5 CONCLUSION

During submaximal steady‐state exercise, adolescent subjects had smaller V_T_/FVC, greater BF, and demonstrated a more variable and random ventilatory response compared to adults. During adolescence, the control of exercise ventilatory response is challenged by changing respiratory and central nervous system hormonal, psychosocial, and systemic characteristics. It is proposed that the greater variability and randomness of the ventilatory response during exercise observed in the adolescent subjects possibly reflects the plasticity and modulation of exercise ventilatory control during the transition from childhood to adulthood and should be viewed as normal.

## AUTHOR CONTRIBUTIONS

**Ryan Welch:** Conceptualization; data curation; formal analysis; investigation; methodology; project administration. **Sarah Casey:** Data curation; investigation. **John Kolbe:** Conceptualization; methodology; supervision. **Kevin Ellyett:** Conceptualization; data curation; formal analysis; supervision.

## CONFLICT OF INTEREST STATEMENT

The authors declare no conflicts of interest.

## Data Availability

Data available upon request to the corresponding author.

## References

[phy270846-bib-0001] Bansal, T. , Haji, G. S. , Rossiter, H. B. , Polkey, M. I. , & Hull, J. H. (2018). Exercise ventilatory irregularity can be quantified by approximate entropy to detect breathing pattern disorder. Respiratory Physiology & Neurobiology, 255, 1–6. 29730423 10.1016/j.resp.2018.05.002

[phy270846-bib-0002] Barstow, T. J. (1994). Characterization of VO_2_ kinetics during heavy exercise. Medicine and Science in Sports and Exercise, 26(11), 1327–1334. 7837952

[phy270846-bib-0003] Becklake, M. R. , & Kauffmann, F. (1999). Gender differences in airway behaviour over the human life span. Thorax, 54(12), 1119–1138. 10567633 10.1136/thx.54.12.1119 PMC1763756

[phy270846-bib-0004] Bien, M. , Hseu, S. , Yien, H. , Kuo, B. I. , Lin, Y. , & Wang, J. (2004). Breathing pattern variability: A weaning predictor in postoperative patients recovering from systemic inflammatory response syndrome. Intensive Care Medicine, 30(2), 241–247. 14647889 10.1007/s00134-003-2073-8

[phy270846-bib-0005] Boulding, R. , Stacey, R. , Niven, R. , & Fowler, S. J. (2016). Dysfunctional breathing: A review of the literature and proposal for classification. European Respiratory Review, 25(141), 287–294. 27581828 10.1183/16000617.0088-2015 PMC9487208

[phy270846-bib-0006] Brennan, M. , Palaniswami, M. , & Kamen, P. (2001). Do existing measures of Poincare plot geometry reflect nonlinear features of heart rate variability? IEEE Transactions on Biomedical Engineering, 48(11), 1342–1347. 11686633 10.1109/10.959330

[phy270846-bib-0007] Brischetto, M. J. , Millman, R. P. , Peterson, D. D. , Silage, D. A. , & Pack, A. I. (1984). Effect of aging on ventilatory response to exercise and CO2. Journal of Applied Physiology (1985), 56(5), 1143–1150. 10.1152/jappl.1984.56.5.1143 6427148

[phy270846-bib-0008] Caldirola, D. , Bellodi, L. , Caumo, A. , Migliarese, G. , & Perna, G. (2004). Approximate entropy of respiratory patterns in panic disorder. The American Journal of Psychiatry, 161(1), 79–87. 14702254 10.1176/appi.ajp.161.1.79

[phy270846-bib-0009] Cooper, D. M. , Kaplan, M. R. , Baumgarten, L. , Weiler‐Ravell, D. , Whipp, B. J. , & Wasserman, K. (1987). Coupling of ventilation and CO2 production during exercise in children. Pediatric Research, 21(6), 568–572. 3110725 10.1203/00006450-198706000-00012

[phy270846-bib-0010] DeGroodt, E. G. , van Pelt, W. , Borsboom, G. J. , Quanjer, P. H. , & van Zomeren, B. C. (1988). Growth of lung and thorax dimensions during the pubertal growth spurt. The European Respiratory Journal, 1(2), 102–108. 3360086

[phy270846-bib-0011] Dejours, P. (1975). Principles of Comparative Respiratory Physiology. North‐Holland Publishing Company.

[phy270846-bib-0012] Fernandes, M. V. S. , Muller, P. D. T. , Santos, M. C. D. , Silva, W. A. D. , Chiappa, A. M. G. , & Chiappa, G. R. (2022). Ventilatory Variability during Cardiopulmonary Exercise Test Is Higher in Heart Failure and Chronic Obstructive Pulmonary Disease plus Heart Failure than in Chronic Obstructive Pulmonary Disease Patients. Journal of Cardiovascular Medicine, 23(10), 694–969. 36099077 10.2459/JCM.0000000000001327

[phy270846-bib-0013] Fletcher, G. F. , Balady, G. J. , Simons‐Morton, D. , Williams, M. A. , Bazzarre, T. , & Amsterdam, E. A. (2001). Exercise standards for testing and training: A statement for healthcare professionals from the American Heart Association. Circulation, 104(14), 1694–1740. 11581152 10.1161/hc3901.095960

[phy270846-bib-0014] Giardini, A. , Odendaal, D. , Khambadkone, S. , & Derrick, G. (2011). Physiologic decrease of ventilatory response to exercise in the second decade of life in healthy children. The American Heart Journal, 161(6), 1214–1219. 21641371 10.1016/j.ahj.2011.03.008

[phy270846-bib-0015] Gomes, N. S. , Silva, W. A. , Pena, R. , & Chiappa, G. R. (2023). Heliox improves minute‐ventilation variability during incremental maximal exercise in COPD patients. Revista Terapia Manual, 21, 1–7.

[phy270846-bib-0016] Graham, B. L. , Steenbruggen, I. , Miller, M. R. , Barjaktarevic, I. Z. , Cooper, B. G. , Hall, G. L. , Hallstrand, T. S. , Kaminsky, D. A. , McCarthy, K. , McCormack, M. C. , Oropez, C. E. , Rosenfeld, M. , Stanojevic, S. , Swanney, M. P. , & Thompson, B. R. (2019). Standardization of spirometry 2019 update. An official American Thoracic Society and European Respiratory Society technical statement. American Journal of Respiratory and Critical Care Medicine, 200(8), e70–e88. 31613151 10.1164/rccm.201908-1590ST PMC6794117

[phy270846-bib-0017] Hibbert, M. , Lannigan, A. , Raven, J. , Landau, L. , & Phelan, P. (1995). Gender differences in lung growth. Pediatric Pulmonology, 19(2), 129–134. 7659468 10.1002/ppul.1950190208

[phy270846-bib-0018] Hsu, C. , Tsai, M. , Huang, G. , Lin, T. , Chen, K. , & Ho, S. (2012). Poincaré plot indexes of heart rate variability detect dynamic autonomic modulation during general anesthesia induction. Acta Anaesthesiologica Taiwanica, 50(1), 12–18. 22500908 10.1016/j.aat.2012.03.002

[phy270846-bib-0019] Khoo, M. C. K. (2000). Determinants of ventilatory instability and variability. Respiration Physiology, 122(2), 167–182. 10967342 10.1016/s0034-5687(00)00157-2

[phy270846-bib-0020] Leung, J. , Kosinski, P. D. , Croal, P. L. , & Kassner, A. (2016). Developmental trajectories of cerebrovascular reactivity in healthy children and young adults assessed with magnetic resonance imaging. Journal of Physiology, 594(10), 2681–2689. 26847953 10.1113/JP271056 PMC4865568

[phy270846-bib-0021] Merkus, P. J. , Borsboom, G. J. , Van Pelt, W. , Schrader, P. C. , Van Houwelingen, H. C. , & Kerrebijn, K. F. (1993). Growth of airways and air spaces in teenagers is related to sex but not to symptoms. Journal of Applied Physiology, 75(5), 2045–2053. 8307858 10.1152/jappl.1993.75.5.2045

[phy270846-bib-0022] Mitchell, G. S. , & Babb, T. G. (2006). Layers of exercise hyperpnea: Modulation and plasticity. Respiratory Physiology & Neurobiology, 151(2), 251–266. 16530024 10.1016/j.resp.2006.02.003

[phy270846-bib-0023] Nagano, Y. , Baba, R. , Kuraishi, K. , Yasuda, T. , Ikoma, M. , Nishibata, K. , Yokota, M. , & Nagashima, M. (1998). Ventilatory control during exercise in normal children. Pediatric Research, 43(5), 704–707. 9585019 10.1203/00006450-199805000-00021

[phy270846-bib-0024] Nourry, C. , Deruelle, F. , Fabre, C. , Baquet, G. , Bart, F. , & Grosbois, J. (2005). Exercise flow‐volume loops in prepubescent aerobically trained children. Journal of Applied Physiology, 99(5), 1912–1921. 16002774 10.1152/japplphysiol.00323.2005

[phy270846-bib-0025] Ogoh, S. (2019). Interaction between the respiratory system and cerebral blood flow regulation. Journal of Applied Physiology (1985), 127(5), 1197–1205. 10.1152/japplphysiol.00057.2019 30920887

[phy270846-bib-0026] Pincus, S. M. (1991). Approximate entropy as a measure of system complexity. Proceedings of the National Academy of Sciences‐PNAS, 88(6), 2297–2301. 10.1073/pnas.88.6.2297 PMC51218 11607165

[phy270846-bib-0027] Quanjer, P. , Stanojevic, S. , Cole, T. J. , Baur, X. , Hall, G. L. , & Culver, B. H. (2012). Multi‐ethnic reference values for spirometry for the 3‐95‐yr age range: The global lung function 2012 equations. The European Respiratory Journal, 40(6), 1324–1343. 22743675 10.1183/09031936.00080312 PMC3786581

[phy270846-bib-0028] Quanjer, P. H. , Stanojevic, S. , Golsjan, M. , Brunekreef, B. , Al‐Rawas, O. , & Kuhr, J. (2010). Changes in the FEV1/FVC ratio during childhood and adolescence: An intercontinental study. European Respiratory Journal, 36(6), 1391–1399. 20351026 10.1183/09031936.00164109

[phy270846-bib-0029] Rosenthal, M. , & Bush, A. (2002). The growing lung: Normal development, and the long‐term effects of pre‐ and postnatal insults. European Respiratory Monograph, 19, 1–24.

[phy270846-bib-0030] Samaranayake, C. B. , Warren, C. , Rhamie, S. , Haji, G. , Wort, S. J. , Price, L. C. , McCabe, C. , & Hull, J. H. (2023). Chaotic breathing in post‐COVID‐19 breathlessness: A key feature of dysfunctional breathing can be characterised objectively by approximate entropy. ERJ Open Research, 9(4), 117. 10.1183/23120541.00117-2023 PMC10276923 37362883

[phy270846-bib-0031] Satti, R. , Abid, N. , Bottaro, M. , De Rui, M. , Garrido, M. , & Raoufy, M. R. (2019). The application of the extended Poincaré plot in the analysis of physiological variabilities. Frontiers in Physiology, 10, 116. 30837892 10.3389/fphys.2019.00116 PMC6390508

[phy270846-bib-0032] Sheel, A. W. , Richards, J. C. , Foster, G. E. , & Guenette, J. A. (2004). Sex differences in respiratory exercise physiology. Sports Medicine (Auckland), 34(9), 567–579. 10.2165/00007256-200434090-00002 15294007

[phy270846-bib-0033] Soni, R. , & Muniyandi, M. (2019). Breath rate variability: A novel measure to study the meditation effects. International Journal of Yoga, 12(1), 45–54. 30692783 10.4103/ijoy.IJOY_27_17 PMC6329220

[phy270846-bib-0034] Springer, C. , Barstow, T. J. , & Cooper, D. M. (1989). Effect of hypoxia on ventilatory control during exercise in children and adults. Pediatric Research, 25(3), 285–290. 2704597 10.1203/00006450-198903000-00016

[phy270846-bib-0035] Talbot, J. S. , Perkins, D. R. , Tallon, C. M. , Dawkins, T. G. , Douglas, A. J. M. , Beckerleg, R. , Crofts, A. , Wright, M. E. , Davies, S. , Steventon, J. J. , Murphy, K. , Lord, R. N. , Pugh, C. J. A. , Oliver, J. L. , Lloyd, R. S. , Ainslie, P. N. , McManus, A. M. , & Stembridge, M. (2023). Cerebral blood flow and cerebrovascular reactivity are modified by maturational stage and exercise training status during youth. Experimental Physiology, 108(12), 1500–1515. 37742137 10.1113/EP091279 PMC10988468

[phy270846-bib-0036] Tallon, C. M. , Barker, A. R. , Nowak‐Flück, D. , Ainslie, P. N. , & McManus, A. M. (2020). The influence of age and sex on cerebrovascular reactivity and ventilatory response to hypercapnia in children and adults. Experimental Physiology, 105(7), 1090–1101. 32333697 10.1113/EP088293

[phy270846-bib-0037] Tarumi, T. , & Zhang, R. (2018). Cerebral blood flow in normal aging adults: Cardiovascular determinants, clinical implications, and aerobic fitness. Journal of Neurochemistry, 144(5), 595–608. 28986925 10.1111/jnc.14234 PMC5874160

[phy270846-bib-0038] Veiga, J. , Lopes, A. J. , Jansen, J. M. , & Melo, P. L. (1985). Airflow pattern complexity and airway obstruction in asthma. Journal of Applied Physiology, 111(2), 412–419. 10.1152/japplphysiol.00267.2011 21565988

[phy270846-bib-0039] Welch, R. , Kolbe, J. , Lardenoye, M. , & Ellyett, K. (2021). Novel application of Poincaré analysis to detect and quantify exercise oscillatory ventilation. Physiological Measurement, 42(4), 04NT01. 10.1088/1361-6579/abf05d 33740782

[phy270846-bib-0040] Weston, M. E. , Barker, A. R. , Tomlinson, O. W. , Coombes, J. S. , Bailey, T. G. , & Bond, B. (2021). Differences in cerebrovascular regulation and ventilatory responses during ramp incremental cycling in children, adolescents, and adults. Journal of Applied Physiology, 131(4), 1200–1210. 34435503 10.1152/japplphysiol.00182.2021

[phy270846-bib-0041] Yentes, J. M. , Hunt, N. , Schmid, K. K. , Kaipust, J. P. , McGrath, D. , & Stergiou, N. (2013). The appropriate use of approximate entropy and sample entropy with short data sets. Annals of Biomedical Engineering, 41(2), 349–365. 23064819 10.1007/s10439-012-0668-3 PMC6549512

[phy270846-bib-0042] Yeragani, V. K. , Radhakrishna, R. K. A. , Tancer, M. , & Uhde, T. (2002). Nonlinear measures of respiration: Respiratory irregularity and increased chaos of respiration in patients with panic disorder. Neuropsychobiology, 46(3), 111–120. 12422057 10.1159/000066388

